# Bloodstream form pre-adaptation to the tsetse fly in *Trypanosoma brucei*

**DOI:** 10.3389/fcimb.2013.00078

**Published:** 2013-11-14

**Authors:** Eva Rico, Federico Rojas, Binny M. Mony, Balazs Szoor, Paula MacGregor, Keith R. Matthews

**Affiliations:** Centre for Immunity, Infection and Evolution, School of Biological Sciences, University of EdinburghEdinburgh, UK

**Keywords:** Trypanosoma, differentiation, slender, stumpy, signaling, gene expression, drug-screening

## Abstract

African trypanosomes are sustained in the bloodstream of their mammalian hosts by their extreme capacity for antigenic variation. However, for life cycle progression, trypanosomes also must generate transmission stages called stumpy forms that are pre-adapted to survive when taken up during the bloodmeal of the disease vector, tsetse flies. These stumpy forms are rather different to the proliferative slender forms that maintain the bloodstream parasitaemia. Firstly, they are non proliferative and morphologically distinct, secondly, they show particular sensitivity to environmental cues that signal entry to the tsetse fly and, thirdly, they are relatively robust such that they survive the changes in temperature, pH and proteolytic environment encountered within the tsetse midgut. These characteristics require regulated changes in gene expression to pre-adapt the parasite and the use of environmental sensing mechanisms, both of which allow the rapid initiation of differentiation to tsetse midgut procyclic forms upon transmission. Interestingly, the generation of stumpy forms is also regulated and periodic in the mammalian blood, this being governed by a density-sensing mechanism whereby a parasite-derived signal drives cell cycle arrest and cellular development both to optimize transmission and to prevent uncontrolled parasite multiplication overwhelming the host. In this review we detail recent developments in our understanding of the molecular mechanisms that underpin the production of stumpy forms in the mammalian bloodstream and their signal perception pathways both in the mammalian bloodstream and upon entry into the tsetse fly. These discoveries are discussed in the context of conserved eukaryotic signaling and differentiation mechanisms. Further, their potential to act as targets for therapeutic strategies that disrupt parasite development either in the mammalian bloodstream or upon their transmission to tsetse flies is also discussed.

## Introduction

“This species …..is characterised by showing two distinct forms-the long and slender, and the short and stumpy. These are not sharply divided from one another, but are interconnected by intermediate forms, so as to form an unbroken series, or curve, from the shortest to the longest.”

(Bruce et al., 1912)

“It has now been shown that the short forms are definitely capable of surviving in the transmitting host, but that does not in itself exclude the intermediate and long forms from a similar development. This apparently does not occur.”

“The shorter forms are those destined to carry on the cycle in the transmitting host.”

(Robertson, 1912)

**Figure 1:**
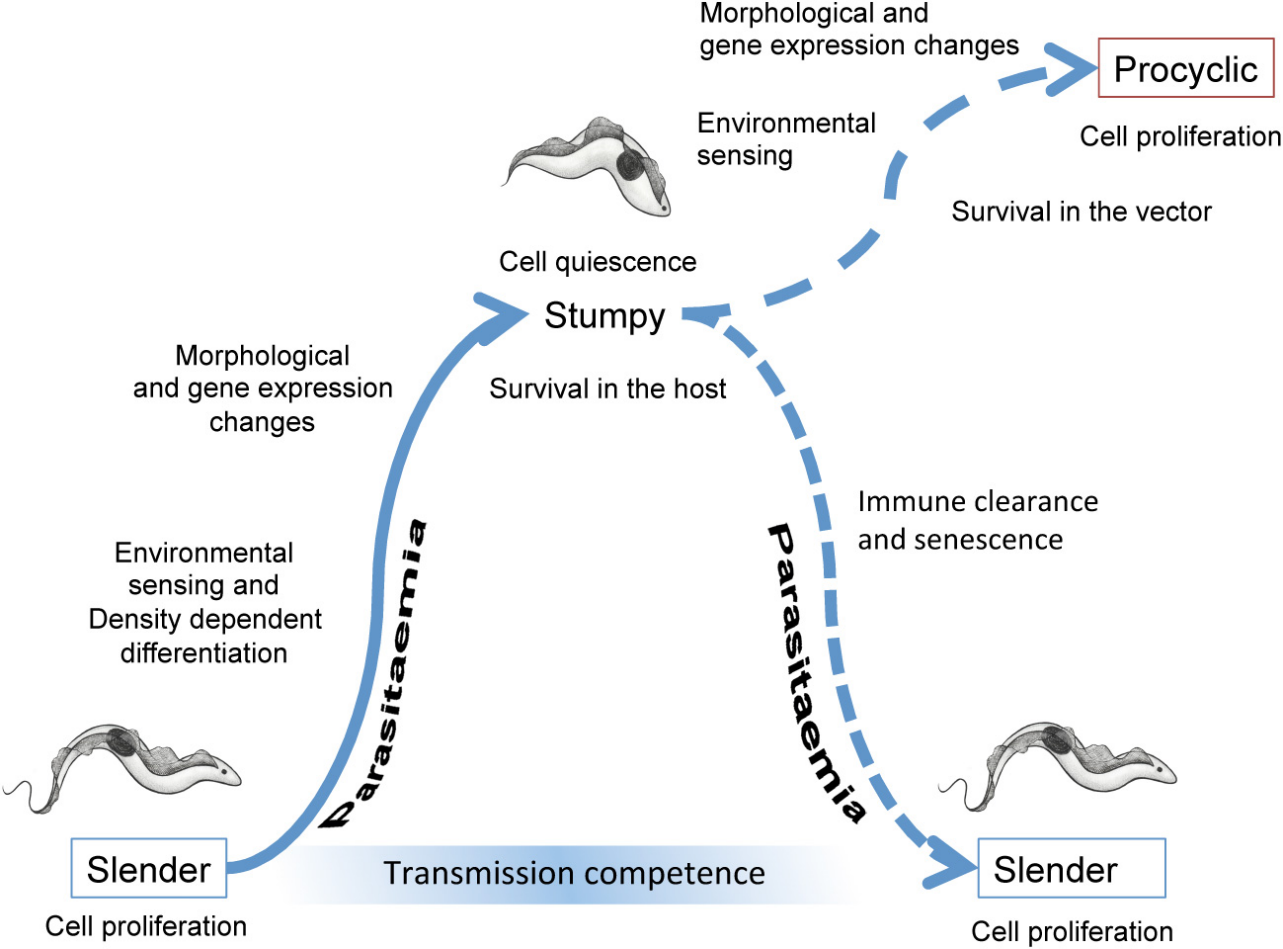
**Events accompanying the development of trypanosomes in their mammalian host and upon transmission to tsetse flies.** In the bloodstream, slender forms proliferate to increase the parasitaemia. Cell density is perceived via the release and detection of stumpy induction factor, this leading to changes in the morphology and gene expression of the parasites as they undergo development to stumpy forms. The stumpy forms are adapted to short term survival in the blood and transmission to tsetse flies. When taken up in a bloodmeal, stumpy forms perceive their new environment and then undergo morphological, metabolic and gene expression changes as they differentiate to procyclic forms. These colonize the tsetse midgut where, after further development and migration to the tsetse salivary glands, they become infective to new mammalian hosts.

The presence of distinct morphological forms in the bloodstream of mammalian hosts infected with African trypanosomes was recognized very early in the research into these parasites and the link between this transition and disease transmission proposed soon after (Robertson, 1912). However, the connection between the different forms in the blood, termed “slender” and “stumpy” at their morphological extremes, and parasite transmission potential remained controversial for nearly a century thereafter. This was contributed to by the fact that the serial laboratory passage of trypanosomes through rodent hosts prior to the development of cryopreservation methods in the late 1950–1960s selected against the development of stumpy forms. This was because stumpy forms are irreversibly arrested in their cell cycle (Shapiro et al., 1984) and require uptake by tsetse flies in order to recommence cell division as midgut procyclic forms. The resulting laboratory-adapted “monomorphic” forms are similar in appearance to the slender-intermediate forms in natural infections and can differentiate to procyclic forms in culture if exposed to high concentrations of citrate/*cis*-aconitate (CCA; Brun and Schonenberger, 1981; Roditi et al., 1989). This complicated the interpretation of studies analysing the relative importance of stumpy forms in transmission and it is only over the last 15 years that the particular characteristics of stumpy forms that adapt them for transmission have been recognized [reviewed in MacGregor et al. (2012)]. This has been enabled by methods for the culture and transfection of pleomorphic trypanosomes (i.e., those capable of stumpy formation) (Reuner et al., 1997; Engstler and Boshart, 2004) and the availability of tools to analyse development of trypanosomes in the bloodstream quantitatively and in molecular detail (Reuner et al., 1997; Dean et al., 2009; MacGregor et al., 2011). All of these studies have reemphasized that stumpy forms fulfil a crucial role in the life-cycle of the trypanosomes, assisting both infection chronicity and transmission potential. Both of these components are linked, since the development of arrested stumpy forms in preparation for transmission also limits the ascending parasitaemia, prolonging host survival (Figure 1). This provides an elegant situation whereby the parasite creates an infection dynamic that balances parasite survival through immune evasion, host survival through restricted parasite virulence and an adaptation for transmission and establishment in the tsetse fly midgut (Lythgoe et al., 2007; Gjini et al., 2010; MacGregor et al., 2011, 2012). This infection dynamic is established by the ability of the trypanosomes to monitor their density in the mammalian bloodstream using a system in some ways analogous to the quorum sensing (QS) systems employed by bacteria. However, whilst those systems are often exploited by microbes to share resources or to co-operate to invade or colonize their current environment (Diggle et al., 2007), trypanosomes are also exploiting environmental information to prepare for their colonization of a new environment.

In this review we detail the several mechanisms and processes that trypanosomes exploit to ensure their capacity for transmission through the development of stumpy forms. We firstly discuss the recent analyses of gene expression and protein changes that accompany the transition from proliferative slender forms to arrested stumpy forms. Secondly, we describe how the parasite regulates its production of transmission stages by density sensing mechanisms in the mammalian bloodstream. Thirdly, we detail how the parasite surface is adapted for environmental sensing and survival and for transmission of the parasite and, fourthly, how entry into the tsetse midgut is perceived by the parasite and the signal transduced to initiate the development to procyclic forms. Finally, we discuss how the developmental transitions that the trypanosome undergoes in preparation for, or during, transmission can be targeted in therapeutic approaches that act both to limit virulence and to prevent disease spread.

## Gene expression regulation during the slender to stumpy transition as a pre-adaptation for differentiation

Overlaid onto the organization of the African trypanosome genome into multi-gene transcription units (Clayton, 2002) is its developmental regulation, with large scale changes in gene expression being observed during the parasite life cycle in order to adapt to the different conditions encountered in the mammal and insect environments. Recently, three different studies analysed the expression profile of mRNAs between bloodstream forms and procyclic forms utilizing pleomorphic slender forms, *in vivo* generated stumpy forms (differentiated from slender forms) and cells undergoing synchronous differentiation from stumpy to procyclic forms (Jensen et al., 2009; Kabani et al., 2009; Nilsson et al., 2010). Despite the different approaches used by the three laboratories [i.e., microarrays (Jensen et al., 2009; Kabani et al., 2009) vs. spliced leader trapping (Nilsson et al., 2010) or biological samples obtained from immunocompromised mice (Kabani et al., 2009; Nilsson et al., 2010) vs. rats (Jensen et al., 2009)], generally complementary results were achieved showing that, contrary to what had been previously suggested, a quite high percentage of *T. brucei* genes are differentially expressed between developmental forms in the blood and the tsetse midgut [25–40% of all genes, according to Jensen et al. (2009); Nilsson et al. (2010)]. Within these studies a number of group comparisons generated outcomes relevant to the developmental or environmental regulation of gene expression in the parasites. For example, Jensen et al. observed, surprisingly, that cultured bloodstream forms and *in vivo* derived slender forms showed similar expression profiles despite their predicted biological differences, whereas the distinctions between logarithmic and stationary procyclic forms were more pronounced (Jensen et al., 2009). Moreover, from all three studies it was clear that the stumpy forms constitute a pre-adaptation required for transmission, with these cells having already undergone many changes in expression when compared to slender forms.

Overall, the most highly regulated transcripts between slender and stumpy forms are those involved in metabolism, cell structure, protein transport, proteolysis and translation, as well as many transcripts with currently unknown function. As expected, slender forms express significantly more ESAGs and GRESAGs (both usually, but not always, found in telomeric and subtelomeric regions together with the VSGs) than stumpy forms (Kabani et al., 2009), with the exception of several ESAG9s that are known to be up-regulated in stumpy forms and have been suggested to be involved in interaction with the external environment (Barnwell et al., 2010). Consistent with slender forms being proliferative and stumpy forms being cell-cycle arrested quiescent forms, histones, DNA replication/repair and translation-related transcripts were found to be up-regulated in slender compared to stumpy forms (Kabani et al., 2009). Other transcripts showing higher expression in the proliferative forms included those associated with the cytoskeleton, especially those related to the flagellum, as well as transcripts involved in metabolism, predominantly those which are localized to the glycosome or are involved in glucose uptake and glycolysis (i.e., glucose transporter THT1 and PGKC). Contrasting with this, procyclins and PAD (*Proteins Associated with Differentiation*) family members involved in CCA-mediated differentiation, (Dean et al., 2009) are up-regulated in stumpy forms as is MSP-B, associated with the VSG cleavage necessary for the change of surface coat upon differentiation to the insect form (Gruszynski et al., 2006). Further, transcripts involved in cytoskeletal remodeling and membrane protein and lipid biosynthesis are increased in stumpy forms, perhaps contributing to the more robust characteristics of the transmissible forms (Nolan et al., 2000) and their need for rapid membrane rearrangement upon entry into the tsetse fly. It has also been known for some time that lysosome activity is enhanced in stumpy forms (Brickman and Balber, 1994), so the observed up-regulation of a chloride channel transcript in these forms (Kabani et al., 2009) could be explained by its role in the maintenance of lysosomal pH. Other transcripts elevated in stumpy forms are RNA helicases related to RNA processing and two zinc-finger proteins that have been demonstrated to be important in differentiation control (Hendriks et al., 2001; Hendriks and Matthews, 2005; Paterou et al., 2006) and that are apparently involved in the regulation of transmission stage transcripts (Walrad et al., 2012). Finally, metabolic enzymes, such as trypanosome alternative oxidase and fructose-2, 6-biphosphatase, as well as components of the cytochrome oxidase complex and other mithochondrial genes such as ATP synthase subunits and NADH dehydrogenase subunits are up-regulated in stumpy forms (Jensen et al., 2009; Kabani et al., 2009; Nilsson et al., 2010). All of these observations confirm the pre-adaptation of stumpy forms to the more complex metabolic requirements of life in the tsetse fly, which involves a well developed mitochondrion with functional Krebs cycle enzymes, respiratory chain and oxidative phosphorylation enzymes and an increased reliance on the oxidation of amino acids like proline. More recently, an analysis of the transcriptome of slender, intermediate and stumpy forms (Capewell et al., 2013) has shown that many of the changes observed between slender and stumpy forms are already present in the intermediate forms, highlighting that the preparation for transmission occurs early in the development of stumpy forms in the bloodstream, long before the dramatic environmental changes that accompany entry into the tsetse midgut are encountered. This developmental programming is therefore, a response to the parasites own density-sensing signal, and not a predominently environmental response, such as could be generated by the thermal stress or glucose depletion that occurs upon tsetse uptake.

As well as during the transition from slender to stumpy forms, transcriptome changes accompany the synchronous differentiation from stumpy forms to procyclic forms (Kabani et al., 2009; Queiroz et al., 2009). For example the transcripts for ESAG2, ESAG11 and the glucose transporter THT1 are rapidly and progressively down-regulated, whilst EP2 and EP3 procyclin transcripts are up-regulated, preparing the surface coat for the insect stage. Re-entry into DNA synthesis around 8–10 h through differentiation (Matthews and Gull, 1994) is preceded by the induction of histone mRNAs 6 h after exposure to CCA. The activation of translation between 1 and 6 h (Capewell et al., 2013) is also accompanied by an enrichment of mRNAs for different nucleolar proteins and ribosomal components (re-entry into proliferation occurs at 8–19 h into the differentiation programme). The metabolic adaptation to the procyclic form is also confirmed by down-regulation of bloodstream form enriched phosphoglycerate kinase C mRNA and the up regulation of the procyclic form enriched PGKB (Gibson et al., 1988), whilst mitochondrial activation is accompanied by the up-regulation of the components of the cytochrome-oxidase complex soon after the initiation of the differentiation process (Kabani et al., 2009).

From all these studies, it seems clear that changes during the life cycle are accompanied by significant changes in gene expression at the mRNA level, but what is happening to the corresponding proteome? Here the changes appear qualitatively and quantitaviely different, with protein levels also showing a greater amplitude of change during development. A recent study by Gunasekera *et al*. used a combination of SILAC (Stable Isotope Labelling of Amino acids in Cell culture) and mass spectrometry to determine the differences in protein abundance between slender, stumpy and procyclic forms (Gunasekera et al., 2012). The study was able to detect 30% of the total proteome and, although there was some bias against very small proteins, proteins containing more than one transmembrane domain or very basic proteins, achieved good coverage across the genome. An interesting feature of this study was that around 30% (depending on expression parameters) of genes showed relatively low correlation between RNA and protein levels, even for some transcripts with a robust expression profile. This contrasts with a similar SILAC analysis of bloodstream and procyclic forms carried out by Urbaniak et al. (2012), that found a good correlation with transcriptome profiles (albeit with a greater dynamic range in the proteome studies). Nonetheless, analysis of the three life cycle stages (slender, stumpy, procyclic) by Gunasekera *et al*. showed major protein changes during the transition from slender to stumpy forms, with more than 500 proteins being increased in stumpy forms. Of these, proteins showing ≥2-fold increase in stumpy forms belonged mainly to the GO (Gene Ontology) terms: plasma membrane, peroxisome, nucleus, microtubule, lysosome, glycosome, and mitochondrion. Supporting the identified changes, this study showed good correlation with previous data for the levels of the bloodstream form enriched alternative terminal oxidase (TAO) (Chaudhuri et al., 1995) and for the stumpy-specific PAD1 transporter family involved in parasite differentiation (Dean et al., 2009). Moreover, the assembly of oxidative phosphorylation complexes III and IV was suggested as one of the final activation steps for energy production in the insect form, being present only in procyclic forms, whilst complexes I, II, and V were already present in stumpy forms. Finally, and supporting previous data obtained with the splice leader trapping approach by the same group (Nilsson et al., 2010), the study showed that one gene can have different protein products differing in their N-terminus, highlighting alternative splicing as a mechanism to create protein diversity.

These proteomic studies are generally in good agreement with a more recent analysis by Digital Serial Analysis of Gene Expression (Digital-SAGE) of the transcripts enriched in the polysomal fraction of slender and stumpy forms (Capewell et al., 2013). This analysis was designed to identify transcripts that might escape the overall translational repression characteristic of stumpy forms, and pointed to mitochondrial, ribonuclear complexes and macromolecular biosynthetic related transcripts being amongst those more elevated during development to stumpy forms. Moreover, the global translational quiescence of stumpy forms was supported by the depletion of mRNAs encoding ribosomal proteins in the stumpy polysomal material. However, only a few of the polysome-enriched stumpy transcripts showed an *in vitro* differentiation phenotype when analysed by genome wide RNAi analysis (Alsford et al., 2011) indicating a role distinct from the process of differentiation *per se*, with an alternative potential role in cell viability or development in the tsetse fly midgut.

Since gene expression in African trypanosomes is regulated at multiple levels (mRNA stability, translation and protein stability), it is important to understand the mechanisms involved in the control of these processes. In trypanosomatids, the developmental regulation of mRNA abundance has been primarily linked to the presence of regulatory sequences and structures located in the 3′untranslated region (3′UTR) (Clayton and Shapira, 2007) but that can also be present in the 5′UTR (Pasion et al., 1996; Mahmood et al., 2001). As mentioned earlier, alternative splicing has been also identified as a further potential regulatory step, with a total of 676 genes being observed to have changes in major splice site usage between developmental forms (Nilsson et al., 2010). These regulatory signals within untranslated regions are then acted upon by RNA binding proteins that operate to positively or negatively regulate the mRNA transcript's expression. Indeed, a large number of potential RNA binding proteins are encoded in the trypanosome genome (De Gaudenzi et al., 2005; Kramer et al., 2010; Kramer and Carrington, 2011). With regards to the control of developmental gene expression, procyclin genes are the best characterized, being regulated during early development in the tsetse midgut (Hehl et al., 1994; Furger et al., 1997; Walrad et al., 2009). These genes have similar, but distinct, 3′UTRs (Vassella et al., 2001a) and their differential expression is controlled by trans-acting proteins that include the small CCCH protein ZFP3, which specifically associates with EP1 and GPEET mRNA, rather than EP2 and EP3 mRNA, and promotes gene expression through association with the translational apparatus (Paterou et al., 2006; Walrad et al., 2009). Interestingly, a global analysis of the other mRNAs that are also stabilized by ZFP3 showed enrichment for mRNAs that were predominantly abundant in stumpy forms (Walrad et al., 2012), emphasing the importance of this protein in transmission events.

As highlighted above, there is a global decrease in protein synthesis during the transition from the slender to stumpy form (Brecht and Parsons, 1998; Capewell et al., 2013) and yet a number of genes escape this global repression of expression to be upregulated in stumpy forms. In a search for regulatory regions controlling transmission-stage gene expression Kabani et al. (2009) identified, using an oligonucleotide frequency scoring algorithm, two related hexanucleotide sequences (TCTTAC and TTCTTA) that were over-represented in the 3′UTR of transcripts predicted to be enriched in stumpy forms. Although no functional analysis of these motifs has been performed, the first of these showed a positional bias 150–200 nt downstream from the gene stop codon. To more functionally define regulatory regions, two recent studies have focused on the detailed identification of sequences and structures involved in the control of two validated stumpy-specific transcripts, encoding PAD1 and an ESAG9 copy. Utilizing CAT reporter assays in pleomorphic trypanosomes, it was demonstrated that both *PAD1* and *ESAG9* contain regulatory regions in their 3′UTRs that repress expression in the slender life stage and alleviate repression in the stumpy form (MacGregor and Matthews, 2012; Monk et al., 2013). For *PAD1*, the distal end of the 3′UTR was found to contain an mRNA silencing element that represses both mRNA and protein expression. However, specific deletion of this region did not alleviate protein expression silencing, suggesting the additional involvement of proximal regions within the 3′UTR. This was confirmed by the independent integration of the *PAD1* proximal or distal 3′UTR regions into the 3′UTR of a constitutively expressed gene, which resulted in no repression at the mRNA level for the reporter gene. Nevertheless, repression was observed at the protein level, suggesting that protein control may be more stringent, consistent with the fact that *PAD* mRNA is elevated early in intermediate forms (MacGregor et al., 2011, 2012) whilst protein expression is only found in fully differentiated stumpy forms (Dean et al., 2009). In the *ESAG9* 3′UTR a regulatory region of just 34 nt was defined as a bifunctional element responsible for both positive and negative regulation depending on the developmental stage of the parasite (Monk et al., 2013). This element regulated mRNA levels and, in the context of the complete *ESAG9* 3′UTR, also protein levels. The simplest model for this regulation implicated a negative regulator binding this element in slender forms and repressing gene expression, whilst in stumpy forms this would be replaced by a positive regulator alleviating the repression of gene expression. Unlike *PAD1*, insertion of this short regulatory element into the 3′UTR of a constitutively expressed gene had a strong effect on mRNA only, suggesting that other sequences within the *ESAG9* 3′UTR might also contribute to the regulation of translation, for example by stabilizing the association of positive translational regulators. Hence, whilst no similarity in the sequence elements was found between the *ESAG9* and *PAD1* 3′UTRs, in both cases stringent regulation at both the mRNA and protein level was observed to control their exclusive expression in the stumpy form. This stringency of regulation is likely to be important for ensuring the appropriate timing of the expression of genes involved in cell-cycle arrest or transmission during programmed development from the slender to stumpy form.

## Signaling mechanisms that govern stumpy formation in the mammalian bloodstream

The progression of trypanosome infections in the mammalian host follows a wave-like pattern, at least early on, with the actively dividing slender forms dominating the ascending phase leading up to the peak, followed by G1/G0 arrest and differentiation to the stumpy forms. While the interplay of host immune factors and the antigenic variation of the parasite play a major role in the waves of parasitaemia, the slender to stumpy transition is an important additional contributor (Lythgoe et al., 2007; Gjini et al., 2010; MacGregor et al., 2011), primarily dictated by parasite density (Reuner et al., 1997). Indeed, pleomorphs reach a threshold parasitaemia before they undergo differentiation (Seed and Sechelski, 1989), and *in vitro* this is linked to the parasite number regardless of the culture seeding density (Reuner et al., 1997). Since monomorphic strains continue to grow to higher cell densities under similar growth conditions as the arrested pleomorphs a limitation in medium components seems unlikely and, indeed, nutrient replacement fails to restore the growth of dense pleomorph cultures (Hesse et al., 1995). Instead, early tentative evidence for a secreted factor controlling parasite growth was provided by the observation that plasma from infected animals at peak parasitaemia could inhibit trypanosome proliferation (Seed and Sechelski, 1989), whereas *in vitro* studies provided further support for the accumulation of a parasite-derived product able to induce stumpy formation (Reuner et al., 1997; Vassella et al., 1997). This density dependent transition from long slender forms to short stumpy forms is reminiscent of the QS mechanisms observed in several prokaryotes and essentially comprises three main components- the signaling molecule(s), the receptor(s) that perceive the cue(s) and the downstream modulators that relay the signal, triggering a series of changes that ultimately bring about differentiation.

In the case of African trypanosomes, the cells sense the increase in their numbers and consequently cease cell division. This ensures the parasite numbers do not overwhelm the host, as can occur when infections are initiated with differentiation-incompetent monomorphic lines (Turner et al., 1995). The development of a plate-based technique for growing pleomorphic strains *in vitro* (Vassella and Boshart, 1996), led to the discovery that a soluble, low molecular weight factor, termed Stumpy Induction Factor (SIF) was able to provide the signal for differentiation (Reuner et al., 1997; Vassella et al., 1997), ruling out a major contribution from the host and highlighting the signal as a parasite-intrinsic entity. Although monomorphs are incapable of differentiation, they do produce SIF (Vassella et al., 1997), and are therefore “signal-blind,” a necessary requirement for their selection in a background population of parasites producing SIF (MacGregor et al., 2012).

**Figure 2:**
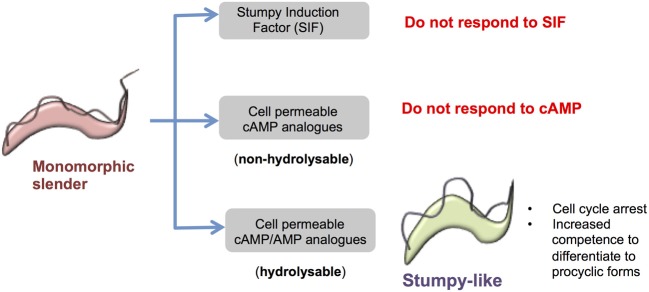
**Response of monomorphic slender cells to cell permeable analogs of cAMP/AMP.** Monomorphic slender cells have lost the capacity to generate stumpy forms *in vivo*, showing a reduced ability to respond to stumpy induction factor. However, when exposed to cell permeable analogs of cAMP or AMP these cells stop proliferation and express some, but not all, characteristics of stumpy forms, namely proliferation arrest, increased capacity for differentiation to procyclic forms and the increased expression of some mRNAs enriched in stumpy forms. This is not a cAMP-mediated response because hydrolysable but not non-hydrolysable cAMP analogs induce the transition and cell permeable AMP is considerably more potent than cell permeable cAMP. Hence the cell permeable cAMP is likely metabolized to cell permeable AMP to elicit the production of stumpy-like forms.

In terms of intracellular signaling, the addition of parasite-conditioned media (containing SIF) causes an increase in cAMP levels in pleomorphs (Vassella et al., 1997). This is consistent with the 2–3-fold increase in intracellular cAMP observed when early log phase slender parasites reach peak parasitaemia in rodents, which is followed by a decrease in the cAMP levels upon appearance of intermediate and stumpy forms (Mancini and Patton, 1981). This suggested cAMP might act as a second messenger conveying the SIF signal. Moreover, further support for a role of cAMP in development was provided by the observation that a cell permeable analog of cAMP (8-pCPT-2′-O-Me-cAMP) promoted G1/G0 arrest in pleomorphs on plates (Vassella et al., 1997), whereas procyclic cells were quite insensitive to high concentrations (up to 1 mM or more) of various cell-permeable cAMP derivatives (Oberholzer et al., 2007). However, unlike *Plasmodium falciparum*, where differentiation to transmissible gametocytes is mediated by cGMP (Hopp et al., 2012), the slender to stumpy transition in trypanosomes now appears not to be a conventional cyclic nucleotide response but is restricted to cell permeable cAMP analogs (Vassella et al., 1997). Furthermore, the hydrolysis resistant analog of cAMP (Sp-8-pCPT-2′-O-Me-cAMPS) is not anti-proliferative for monomorphic slender forms whereas the degradation products of cAMP, namely AMP and adenosine, have a more potent effect, indicating that these molecules are likely to be the key players in the signaling cascade rather than cyclic nucleotides (Laxman et al., 2006) (Figure 2). Supporting this, knockdown of the *T.brucei* PDEB family, which causes an increase in the levels of intracellular cAMP, failed to promote differentiation (Oberholzer et al., 2007) despite the ability of etazolate, a phosphodiesterase (PDE) inhibitor to induce this transition (Vassella et al., 1997), a discrepancy possibly due to the proposed lack of specificity of etazolate for trypanosomal PDEs (Laxman et al., 2006). Further evidence for cAMP not being the direct modulator of differentiation was recently provided by the observation that the levels of *Tb*TOR4 protein (whose loss of function triggers the appearance of stumpy-like forms, see below) decreased when parasites were exposed to 8-pCPT-2′-O-Me-cAMP or 8-pCPT-2′-O-Me-AMP but not Sp-8-pCPT-2′-O-Me-cAMPS (Barquilla et al., 2012). Combined this points to a non-canonical intracellular signaling pathway operating to regulate stumpy formation, that does not involve cAMP as a second messenger.

To date no component of a signaling pathway that has a direct role in *promoting* stumpy formation has been identified. However, genes whose loss of function triggers quiescence (and hence are differentiation *inhibitors*) have provided some molecular insight into the signaling cascade. For example, the deletion of a Zinc finger kinase (ZFK) in pleomorphs reduced growth and also increased the rate of the slender to stumpy transition, albeit only *in vitro* (Vassella et al., 2001b). Similarly, knock out of a mitogen activated protein kinase homolog, *Tb*MAPK5, gave a similar phenotype, with the exception that stumpy formation *in vivo* was also accelerated (Domenicali Pfister et al., 2006). Whilst neither the ZFK nor MAPK knockouts could induce differentiation in monomorphs, deletion of the TOR kinase member, *Tb*TOR4, stimulated monomorphs to generate stumpy-like forms (Barquilla et al., 2012), positioning this molecule downstream of, or on a distinct pathway to, the other inhibitors.

The evidence for the existence of the SIF signaling factor(s) itself remains circumstantial. It is believed to be a small (<500 Da), soluble and filterable factor, distinguishing it from other parasite communication systems, such as the secreted vesicular exosomes recently described for *Plasmodium* parasites (Mantel et al., 2013; Regev-Rudzki et al., 2013). Although the identity of SIF has eluded us to date, a recent intriguing finding on the ability of derivatives of the differentiation inducing factor (DIF) from *Dictyostelium discoideum* to reduce proliferation of *T. cruzi* parasites *in vitro* as well as *in vivo* (Nakajima-Shimada et al., 2013) suggests that there might be some conservation of eukaryotic QS processes. Until recently, one of the major obstacles to determining the molecular nature of SIF was the lack of a robust marker for stumpy formation that could enable high throughput screens for stumpy inducers. However, the discovery of PAD1 as a stumpy-specific marker (Dean et al., 2009) and the development of reporter lines using this marker (MacGregor and Matthews, 2012), now enables activity-guided assays for screening components of conditioned medium, assisting the purification of SIF.

## Adaptations for survival in the mammalian host by stumpy form trypanosomes and signaling events upon transmission

### Surface molecules

Once stumpy forms are generated from slender forms they are irreversibly arrested and have a limited lifespan in the bloodstream (Turner et al., 1995). Nonetheless, to maximize their potential for transmission, stumpy form trypanosomes exhibit a number of adaptations that both prolong their survival in the mammalian bloodstream (increasing their probability of uptake by tsetse flies) and promote their viability upon entering the tsetse midgut.

Perhaps the most obvious survival mechanism employed by trypanosomes in the mammalian blood is their expression of a variant surface glycoprotein, providing protection against the alternative pathway of complement activation (Ferrante and Allison, 1983) and, through antigen variation, antibody-mediated killing. This protection extends only to trypanosomes that have changed their surface antigen coat and is therefore, probably not available to non-proliferating stumpy forms. Nonetheless stumpy forms do show a particular adaptation allowing their preferential survival when exposed to increasing titres of antigen-specific antibody during a wave of infection. This process removes the bound antibodies from the surface of the parasite by using hydrodynamic flow forces (Engstler et al., 2007). Specifically, the flagellum of trypanosomes, attached along the cell body, propels the parasite anteriorly. This causes the movement of bound immune complexes by hydrodynamic drag forces backwards toward the posterior flagellar pocket. Using cell-surface VSG labeled with a membrane-impermeable blue-fluorescent dye, the majority of immunoglobulin bound to VSG was found to be transported to the lysosome, where the immunoglobulin was degraded, whereas the VSG was recycled to the cell surface. The directional movement of the immunoglobulin-VSG complex toward the posterior end of the cell and its subsequent rapid endocytosis provides a very efficient mechanism to clear immune complexes from the cell surface and is twice as rapid for stumpy forms as for slender forms (Engstler et al., 2007). Hence, this phenomenon could contribute to the greater resistance of stumpy forms to antibody-mediated killing than slender forms (McLintock et al., 1993), allowing the stumpy forms to predominate at peak parasitaemia and extending the period at which the infection is transmissible to tsetse flies.

Other molecules at the parasite surface and within the flagellar pocket show developmental regulation during development between slender and stumpy forms. Whilst ESAG9 is upregulated early during stumpy formation, as detailed earlier, the haptoglobin-haemoglobin receptor in the flagellar pocket is downregulated (Vanhollebeke et al., 2010). This molecule is involved in haptoglobin-haemoglobin uptake, which may no longer be needed in the quiescent stumpy form. However, it might also represent a protection mechanism for transmission stages against TLF1, since the haptoglobin haemoglobin receptor is the route for the uptake of the trypanosome lytic factor in human serum (Vanhollebeke et al., 2008). Since the resistance to TLF is mediated in *Trypanosoma brucei rhodesiense* by an expression site component, SRA (Xong et al., 1998), and expression site transcription is strongly down regulated in stumpy forms (Amiguet-Vercher et al., 2004), the downregulation of the haptoglobin-haemoglobin receptor in stumpy forms could prevent TLF1-mediated killing when SRA production is reduced. TLF2-mediated killing would remain possible, however, unless parasites were sequestered in the tissues or lymphatics, as has been suggested for metacyclic forms (Pays et al., 2006).

At the level of mRNA transcripts, the abundant invariant surface glycoproteins (ISGs) 65 and 75 [present at ~70,000 and 50,000 copies, respectively, (Ziegelbauer and Overath, 1992)] are also down regulated in stumpy forms with respect to slender forms (Nilsson et al., 2010). ISG proteins have type I transmembrane topology and intercalate with VSG on the surface (Leung et al., 2011). Although no identified function has been assigned for these surface proteins, ISG75 was recently identified in a genome-wide RNAi-screen to mediate the uptake of the trypanocidal drug suramin (Alsford et al., 2012). ISG65 and ISG75 have been found to be present at the surface and endosomal membranes and are turned over more rapidly than VSGs in a process involving ubiquitination of lysine residues in their cytoplasmic domains. Specifically, ISG65 has been shown to be ubiquitinated on residues K3 and K4 of the cytoplasmic tail, with mutation of these residues significantly increasing the protein half life (Chung et al., 2008). ISG75 is also ubiquitynated in *T. brucei* and the absence of cytoplasmic lysine residues renders the protein incapable of modification by ubiquitin and consequently stabilized (Leung et al., 2011).

As well as proteins at the cell surface membrane and flagellar pocket, it has been hypothesized that the parasite flagellum functions as a signaling organelle for integrating host-derived and parasite-derived signals and these could contribute to development both in the bloodstream and upon transmission to tsetse flies. For example, proteins involved in cyclic nucleotide signaling, Ca^2+^ signaling and phospho-signaling pathways are anchored to the *T. brucei* flagellum skeleton (Wu et al., 1994; Oberholzer et al., 2007; Smith et al., 2009). *T. brucei* calflagins Tb17, Tb24, Tb44 are all calcium sensors that associate with the flagellar membrane in a calcium-dependent and palmitoylation-dependent manner, and although their precise function its not clear, a role for these proteins in host survival and immune evasion has been described (Emmer et al., 2010). Monomorphic calflagin-deficient parasites exhibit a normal growth rate *in vivo* during the first week post-infection, but subsequently parasite numbers decrease to undetectable levels, followed by the emergence of a parasite population expressing alternative VSGs. This phenotype does not involve any failure in surface-bound antibody clearance or parasite motility, indicating that calflagins could promote parasite resistance to early host immunity. In a recent study, by performing flagellum purification, combined with affinity purification of surface exposed proteins and multidimensional protein identification technology (MudPIT), many novel and already identified proteins were found on the flagellum surface (Oberholzer et al., 2011). This included receptor-like proteins with a large extracellular domain, that could be suitable for binding host ligands, connected to an intracellular signaling module, for example a kinase domain, supporting the idea that the flagellum might be able to sense the environment and transmit signals inside the cell to assist their adaptation, differentiation or survival in the bloodstream.

### Adenylate cyclase signaling in the mammalian bloodstream

The *T. brucei* genome encodes more than 80 adenylate cyclases (ACs), many of which are at the cell surface (Alexandre et al., 1996; Bridges et al., 2008). The prototype gene of this family, termed ESAG4, is part of VSG expression sites and so bloodstream stage-specific, unlike the family of related genes, GRESAG4 (genes related to ESAG4) which are positioned outwith expression sites and constitutively transcribed throughout the life cycle of the parasite.

The ACs of *T.brucei* share a similar structure, with a large extracellular domain (900 amino acids) and a conserved intracellular catalytic domain (around 350 amino acids) separated by a single transmembrane domain; dimerization of the catalytic domain is required for activity. Although the basal cellular cyclic adenosine monophosphate (cAMP) concentration is extremely low, stress, such as acid treatment or osmotic lysis, activates ACs by up to two orders of magnitude (Rolin et al., 1996, 1998; Nolan et al., 2000). Recently, ESAG4 activity has been shown to be crucial to regulate processes involved in the early establishment of the parasitaemia in the host (Salmon et al., 2012b). By overexpressing a catalytically dead mutant (DNc) of ESAG4, total AC activity was reduced by 50% and transient growth retardation and cytokinesis abnormalities in culture were seen, subsequently compensated by an upregulation of GREG4.1 (Salmon et al., 2012a). In mice, however, the parasitaemia of DNc cells was considerably reduced, and the host survival time was extended. After analysing immune modulators affecting trypanosome infection such as tumor necrosis factor alpha (TNF-α), Interferon gamma (INF-γ), and Nitric oxide (NO), it was shown that TNF-α is crucially involved in controlling the early trypanosome parasitaemia and that the trypanosomal ACs inhibited TNF-α synthesis in liver myeloid cells by activating the myeloid cell PKA (Salmon et al., 2012b). Apparently, therefore, the trypanosome adenylyl cyclases inhibit the innate response of the host by shutting down TNF-α synthesis, helping the infection to establish.

As mentioned earlier, a peak in the activity of AC has also been linked to the transition from the slender form to the stumpy forms (Mancini and Patton, 1981; Vassella et al., 1997); slender cells increase their intracellular cAMP concentration until they reach peak parasitaemia, and then it decreases as the transition to intermediate and short stumpy forms commences. Interestingly, ESAG4 protein levels are reduced in the stumpy form by 93% compared to slender forms, although ablation by RNAi of the ESAG4+ESAG4L subfamily does not modify the slender to stumpy differentiation (Salmon et al., 2012a).

AC activity is also activated by incubation of slender forms at pH 5.5 (perhaps mimicking their activation upon macrophage uptake early in infection), but no similar effect is seen in stumpy forms (Nolan et al., 2000). This correlates with the greater tolerance to high extracellular H^+^ concentration of stumpy forms compared to slender forms, reflecting that stumpy cells are able to maintain a constant cytoplasmic pH. Nonetheless, during the differentiation from stumpy to procyclic forms a significant but transient increase in cyclase activity is observed at around 6 h into differentiation and after the initiation of cell proliferation (Rolin et al., 1998). However, since the first peak in AC activity occurs after the transformation into procyclics begins, it is unlikely that the rise in cAMP generated is involved in the early differentiation events in the tsetse.

### Surface molecules and signaling events as stumpy forms enter the tsetse midgut

There are two known ways that VSG is released from the trypanosome cell surface during differentiation from stumpy to procyclic forms, glycophosphatidyl inositol (GPI) hydrolysis and endoproteolysis. Slender and stumpy trypanosomes contain an endogenous GPI phospholipase C (GPI-PLC) that is capable of hydrolyzing the GPI anchor found on VSG, but not procyclin. GPI-PLC is reported to be located exclusively in a linear array on the outside of the flagellar membrane, close to the flagellar attachment zone (Hanrahan et al., 2009). Deletion of both the GPI-PLC gene and a Zn^2+^-metalloprotease (*Tb*MSP-B) gene prevents the differentiation of monomorphic bloodstream forms into procyclic forms, although the cells remain viable for several days (Grandgenett et al., 2007). In stumpy cells exposed to the differentiation triggers CCA, MSP-B mRNA and protein levels are upregulated, this occurring concurrently with the onset of proteolytic release of VSG (Gruszynski et al., 2006). In contrast, GPI-PLC mRNA levels quickly decrease after differentiation is induced. Protein levels decrease subsequently, although a dramatic loss of enzyme only occurs after the onset of cell division (most probably by cell dilution). Hence the combined action of GPI-PLC and MSP-B operate to enable the rapid and synchronous loss of VSG from differentiating stumpy form trypanosomes that occurs ~6 h after the initiation of differentiation (Gruszynski et al., 2006).

Two genes encoding surface carboxylate transporters, PAD1 and PAD2, have also been shown to be expressed at the surface of stumpy form *T. brucei* and important for the initiation of differentiation. These proteins have differential expression through the life cycle of the parasites, being absent in the replicative slender form and upregulated in the stumpy (PAD1 and PAD2) and procyclic form (PAD2) (Dean et al., 2009). By RNAi targeting both genes it was shown that they are not required for either stumpy formation or survival, but that they are required to perceive CCA at physiological concentrations to enable differentiation from stumpy into procyclic cells. PAD2 was also demonstrated to be released to the cell surface at 20°C, thereby demonstrating a redistribution of the transporter in response to cold shock. The expression profile and differential trafficking of the PAD proteins contributes to appropriate developmental responses in two ways (MacGregor and Matthews, 2010). Firstly, by being absent on slender forms but present on stumpy forms, sensitivity to the differentiation signals is restricted to the transmission-adapted stage. Second, the elevation of PAD2 at low temperature ensures that only those parasites that enter the arthropod midgut can detect and respond to the signal. This signal is then relayed via a phosphatase-signaling cascade to initiate the differentiation events that allow colonization of the tsetse midgut.

## Signal transduction of the differentiation signal upon entry into the tsetse fly

Despite the detailed *in silico* analysis of the TriTryp kinome (Parsons et al., 2005) and phosphatome (Brenchley et al., 2007), a coherent assembly of environmental signaling pathways in trypanosomes is almost entirely missing. One exception is the protein phosphatase cascade that regulates differentiation from bloodstream stumpy forms to vector-adapted procyclic forms.

**Figure 3:**
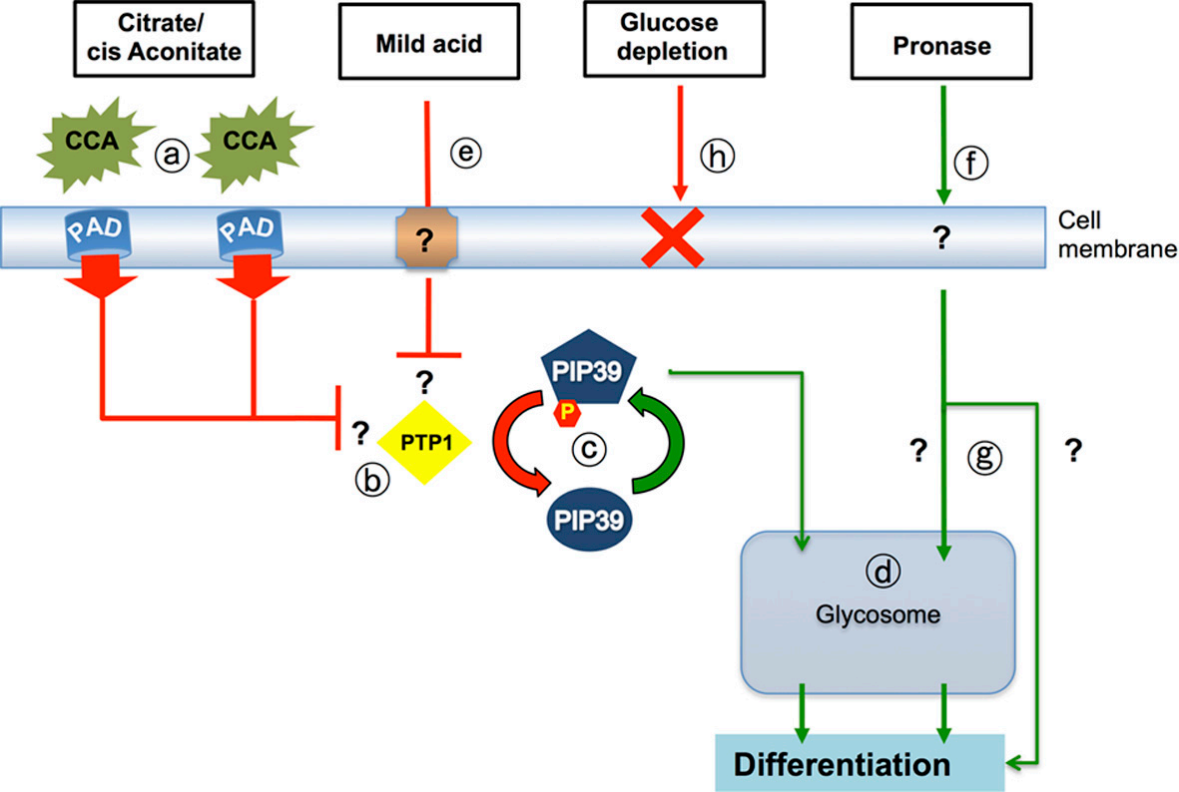
**Signaling pathways that drive differentiation when trypanosomes enter the tsetse midgut.** Citrate-cis aconitate (CCA) is transported by cell membrane PAD proteins (a), this being favored under cold shock conditions. CCA inhibits the activity of the differentiation inhibitor, *Tb*PTP1 (b) a tyrosine phosphatase whose activity is normally enhanced by its substrate, *Tb*PIP39. When *Tb*PTP1 is inhibited, *Tb*PIP39 becomes phosphorylated and more active (c). *Tb*PIP39 can relocate to the glycosome (though this is not dependent upon its phosphorylation) whereupon it acts on unidentified substrates to promote differentiation (d). The same signaling pathway is stimulated by exposure of stumpy forms to mild acid conditions (pH 5.5 for 2 h) (e), which also generates phosphorylated *Tb*PIP39. Exposure of stumpy forms to proteases at the cell surface (f) also stimulates differentiation, but this does not involve *Tb*PTP1 or *Tb*PIP39 and operates through an uncharacterized pathway (g) that may or may not involve the glycosome. It is also not necessary for differentiation in the midgut of tsetse. A reduction of environmental glucose (h) has also been suggested to initiate differentiation, but reducing medium glucose 20-fold, or using the transport inhibitor phloretin, does not cause the differentiation of stumpy forms to procyclic forms.

In stumpy forms, a tyrosine phosphatase (*Tb*PTP1) (Szoor et al., 2006) prevents cells from differentiation until its activity is reduced in the presence of the developmental triggers CCA (Brun and Schonenberger, 1981), whose uptake is controlled by the carboxylate transporter PAD proteins (Dean et al., 2009), as detailed earlier. Recently, a D × D × T phosphatase, *Tb*PIP39, was identified as a downstream regulator of this pathway and shown to be activated upon tyrosine-phosphorylation and, hence, negatively regulated by *Tb*PTP1 (Figure 3). Moreover, the presence of *Tb*PIP39 increased the activity of *Tb*PTP1, reinforcing its own repression and thereby generating a potentially bistable regulatory switch (Szoor et al., 2010). Importantly, this self-repressive loop is interrupted and regulated by CCA, such that differentiation can be initiated. Specifically, in the presence of CCA, the activation of *Tb*PTP1 by *Tb*PIP39 is reduced, such that *Tb*PIP39 becomes rapidly phosphorylated on tyrosine 278, the enzyme activated and differentiation is stimulated (Szoor et al., 2010). RNAi studies using stumpy form cells confirmed that reducing *Tb*PIP39 levels inhibited differentiation, although delayed differentiation remained possible probably through the inability to completely eliminate *Tb*PIP39 from the cells by RNAi. Localization studies showed that *Tb*PIP39 is found in glycosomes, peroxisome-like organelles in trypanosomes, which are the site of glycolysis and several other metabolic activities (Michels et al., 2006). Supporting this, removal of the C-terminal glycosomal localization signal on *Tb*PIP39 prevented its ability to promote differentiation, highlighting that the glycosome is the important site of action for the enzyme. Although the target(s) of *Tb*PIP39 in the glycosome are unknown, as are the kinases that oppose its action, developmental effects mediated through metabolic perturbation are already well known, particularly with respect to their impact on surface protein expression (Morris et al., 2002; Vassella et al., 2004).

Although PAD proteins transport CCA and *Tb*PTP1/*Tb*PIP39 regulatory cross talk is controlled by the levels of these differentiation signals, trypanosomes have also been reported to be able to differentiate to procyclic forms in response to a number of other triggers. These include (i) mild acid treatment (Rolin et al., 1998), (ii) exposure of the parasite surface to limited protease digestion (Yabu and Takayanagi, 1988; Hunt et al., 1994; Sbicego et al., 1999) and (iii) a reduction of glucose, mediated either through the use of glucose-depleted media (Milne et al., 1998), or exposure to phloretin, an inhibitor of glucose uptake (Haanstra et al., 2011). With the identification of phosphorylated *Tb*PIP39 as a signature of differentiation stimulated via *Tb*PTP1, it has been possible to investigate, firstly, the efficiency of each of these reported stimuli on transmissible stumpy forms and, secondly, whether each signal is transduced through the same signaling pathway (Szoor et al., 2013).

These studies revealed that CCA, mild acid and protease treatment of stumpy forms could all stimulate differentiation effectively, but that glucose depletion could not. This was a surprising finding, since glucose levels are believed to rapidly decrease in the tsetse blood meal. With respect to the signaling pathways used, monitoring the phosphorylation of *Tb*PIP39 using a phospho-specific antibody revealed that CCA and mild acid both signal via the *Tb*PTP1/*Tb*PIP39 pathway, whereas protease treatment of the cells does not. Supporting the existence of distinct signaling pathways, ablation of *Tb*PIP39 by RNAi reduced differentiation in response to CCA and mild acid but not protease treatment.

Since protease attack of the parasite surface is believed to occur in the tsetse midgut through the action of trypsin-like activities, the relevance of trypsin like activities in parasite differentiation in the tsetse fly has been recently investigated (Szoor et al., 2013). By feeding stumpy form parasites in the presence of Soybean trypsin inhibitor (STI), tsetse midgut trypsin activity could be almost completely ablated. Despite this, no difference in trypanosome differentiation was observed between control and treated tsetse flies, with equivalent levels of procyclic forms being generated in both populations. This highlighted that trypsin-like activities in the tsetse midgut are not solely responsible for the initiation of differentiation, although a contributory role for these activities remains possible. Supporting this, *in vitro* exposure of bloodstream trypanosomes to both CCA and protease treatment generates a more robust differentiation response than exposure to either trigger alone (Sbicego et al., 1999), suggesting that the two signals could act co-operatively to ensure effective parasite differentiation upon entry to the tsetse fly.

These findings support a central role for the CCA/PAD/*Tb*PTP1/*Tb*PIP39 signaling pathway in regulating differentiation both *in vitro* and *in vivo*, with protease attack of the parasite surface being dispensable for differentiation in the tsetse fly. Apparently, therefore, independent and redundant signaling pathways can operate to control parasite development.

## Phenotypic screening for stumpy formation via small compound screens

As well as providing fundamental biological insight into the developmental pathways operating in these evolutionarily ancient eukaryotes, analyses of the differentiation of trypanosomatids, particularly in the mammalian bloodstream, provides possible drug targets and approaches with the potential to limit the virulence or transmission of these important pathogens. The need for new, safe, efficacious drugs to target African trypanosomiasis is clear (Barrett et al., 2007; Simarro et al., 2012) and considerable efforts are currently underway to identify new therapeutics. The traditional route to identify such therapeutic agents has been to search for compounds that will target genes or pathways necessary for cell viability. Indeed, whole-cell based high-throughput screening for trypanostatic and trypanocidal agents is well established (Hesse et al., 1995; Mackey et al., 2006; Sykes and Avery, 2009; Jones et al., 2010; De Rycker et al., 2012; Sykes et al., 2012; Vodnala et al., 2013) and multiple classes of compounds with potential for development into new therapeutics are being pursued (Mackey et al., 2006; Jones et al., 2010; Sykes et al., 2012; Vodnala et al., 2013). However, phenotypic screens for genes or pathways involved in the establishment, maintenance or transmission of infection could provide an alternative or complementary route to the identification of novel therapeutics with anti-virulence or transmission-blocking potential. Indeed efforts have been made for other pathogens to develop high throughput phenotypic screens whereby small molecules are assessed for their ability to enhance or hinder a biological function, rather than cause direct cytotoxicity. This approach has the additional benefit that compounds perturbing defined cellular processes can provide invaluable tools for pathway dissection.

There are several examples of phenotypic screens that provide a template for dissecting virulence, transmission or other important pathways in pathogens. For example, screens targeting virulence factors in microbes have been used to identify compounds that prevent Type III (Kauppi et al., 2003) or Type IV (Paschos et al., 2011) secretion pathways in pathogenic gram-negative bacteria. Similarly, a compound that reduces adhesion of pathogenic fungi to mammalian epithelial cells was identified through screening for small molecules that block GPI-mannoprotein (e.g., fungal adhesins, where the GPI is removed and the protein linked to β-1,6-glucan) localization to the cell wall (Tsukahara et al., 2003). Such concepts have been also applied to protozoan parasites such as *Toxoplasma gondii* and *Plasmodium falciparum*. During infection, the malaria parasite *P. falciparum* uses multiple pathways for establishment, propagation, and maintenance of the parasitaemia and chemical genetic approaches have aided in the understanding of these processes. For example, a small screen of chemical protease inhibitors targeting the Falcipain-1 protease from cell lysate was then extended in whole cell assays to identify the role of this protease in red blood cell (RBC) invasion by merozoite forms (Greenbaum et al., 2002). This provided both increased understanding of the invasion process as well as a potential new antimalarial able to prevent merozoites from entering RBCs, thereby leaving them exposed to the host immune system. Additionally, protease inhibitors that prevent RBC rupture by *P. falciparum* have been identified through a high-throughput, whole-cell chemical screen, leading to the identification of two proteases, DPAP3 and PfSUB1, which function in release of the parasite from the host cell (Arastu-Kapur et al., 2008). As with *Plasmodium*, the parasite *T.gondii* must also invade host cells in order to establish and maintain an infection, requiring pathways for gliding motility, attachment to the host cell, secretion from the micronemes and invasion. Consequently, *T.gondii* invasion has been the target of multiple whole-cell high throughput small molecule screens (Carey et al., 2004; Hall et al., 2011; Kamau et al., 2012). These have successfully identified compounds targeting different parts of this pathway leading through follow up analysis to improved understanding of myosin motor activity during motility in one case (Heaslip et al., 2010) and to identification of the DJ-1 protein, required for microneme secretion and motility in another (Hall et al., 2011).

Clearly, the developmental biology of trypanosomes in the mammalian bloodstream offers similar possibilities for phenotypic screens. Without the slender form, the trypanosome parasitaemia could not be established or maintained whereas the stumpy form is necessary for cyclical transmission. As such, targeting either stage could eliminate the parasite or block its spread. For example, identification of compounds able to prevent slender cell proliferation or switching of the VSG coat would result in clearance of the parasites by the host immune system. Similarly, identification of compounds that drive proliferative slender forms to non-proliferative stumpy forms, or onward to procyclic forms, if irreversible and complete, would cause clearance of the parasitaemia altogether. Indeed, even if only partial induction of the population was achieved, this could lower the parasitaemia below the threshold needed for tsetse transmission, both reducing virulence and transmission potential possibly providing an adjunctive therapy for conventional trypanocidal drugs. In contrast, compounds able to prevent the slender to stumpy transition, perhaps through blocking of density sensing or operation of the stumpy induction pathway, would result in uncontrolled growth within the host. Whilst this would reduce transmission potential, it would significantly promote virulence and so would not be of therapeutic value.

In order to carry out a phenotypic chemical screen an assay must be designed that allows monitoring of the presence or absence of the phenotype in a simple, cheap and high-throughput manner, often through the use of a marker or reporter protein as a proxy for that phenotype. For developmental studies, molecular markers are known for the different life stages and reporter genes and assays are commonly utilized in the study of trypanosome molecular biology. For example, genetically modified reporter lines that combine a reporter gene with the stumpy-enriched PAD1 or ESAG9 3′UTR have been demonstrated to report on stumpy formation *in vitro* (MacGregor and Matthews, 2012; Monk et al., 2013) and such constructs can be modified in simple assays to screen for chemical inducers of this differentiation event (MacGregor and Matthews, unpublished data). Notwithstanding the caution that the monomorphic cells usually employed in high throughput screens may not reflect the phenotypic responses and drug sensitivities of the more field relevant pleomorphic lines, it is clear that screening for developmental defects in trypanosomes has potential for identifying novel anti-trypanosomal compounds and useful tool compounds for biological research.

## Summary and perspectives

The last few years have provided the first molecular insight into how trypanosomes monitor their environment and prepare for transmission as stumpy forms. Moreover, their environmental signaling pathways are now being uncovered in a coherent analysis that differs from the individual identification of molecules possible through earlier candidate gene analysis. This has been enabled through integration of information provided by high throughput transcriptome and proteomic analyses and through the capacity to extend out from identified nodes where key molecules have been identified using protein interaction studies. However, recently, the development of genome-wide RNAi selection approaches (Alsford et al., 2011, 2012) is providing a step change in the information that is available on molecular processes in trypanosomes. Although initially focused on understanding drug action, these screens have the potential to uncover the molecular components of developmental pathways or gene regulatory networks, particularly where the developmental expression of markers can be exploited to drive selectional screens. To date the application of these screens has been limited to monomorphic lines since their transfection efficiency is higher than the more biologically relevant pleomorphic lines that are crucial to understanding the developmental biology of trypanosomes in their mammalian host. However, as transfection efficiencies improve (MacGregor et al., 2013), RNAi screens will become tractable in pleomorphs and detailed analysis of slender and stumpy form biology will become possible. The application of increased HTS is also a powerful development for both anti-virulence therapies and for dissecting the biology of trypanosome development in its mammalian host. For example, the consequences of particular therapeutic or genetic perturbations can now be analyzed on a genome-wide scale through a combination of transcriptome, proteomic, and phosphoproteomic/post-translational modification analyses. The greatly reduced cost of genome sequencing also now means that different parasite strains can be economically analyzed, such that monomorphic laboratory-adapted lines can be compared with pleomorphic laboratory and field strains, assisting the identification of selected traits that render cells resistant to the stumpy formation signal.

What key aspects of the development of trypanosomes in the mammalian blood remain to be answered? One aspect is the nature of the signal that the trypanosome uses to regulate stumpy formation. Although a SIF or factors has been proposed, it remains the “Loch Ness monster” of trypanosome research, despite 15 years of effort. This failure could reflect several issues. Firstly, until recently, suitable reporter assays were not available to detect SIF activity, restricting the capacity to carry out bioassays. The development of PAD-based reporters to monitor stumpy formation (MacGregor and Matthews, 2012) provides the solution to this problem, applicable also to HTS for anti virulence compounds as detailed above. Secondly, SIF may not be a single entity, but a composite of several co-dependent signals that confound simple purification regimens. In bacterial QS, for example, multiple signals are often used to help the bacterial population perceive cell density in context- for example where there are low or high flow conditions that would change the “interpretation” of a single soluble signal (Diggle et al., 2007). Thirdly, the behavior of pleomorphic cells in culture does not fully reflect their behavior in mice, since parasites grown in liquid culture without methyl cellulose (unlike those on plates) do not form uniformly cell-cycle arrested stumpy forms despite their continued ability to readily undergo this transition *in vivo*. Finally, the evidence for an accumulated signal in stumpy formation remains circumstantial and indirect, such that a perception of depletion of a key environmental component is not yet formally excluded. In each case, the metabolomics-guided refinement of trypanosome culture media and the ability to detect subtle changes in media composition after parasite conditioning by small molecule profiling (Creek et al., 2013) provides one route to narrow down the entities that may control density sensing.

The response of the parasite to density sensing also needs to be dissected in detail. In particular, identification of the molecular components of the SIF reception pathway is tractable and offers the opportunity to generate a coherent picture of a key trypanosome environmental signaling pathway, providing detailed insight into how the parasite interacts with its environment. Upon entering the tsetse fly also, the environmental signaling pathways are being assembled, the link to regulation through the glycosomes (Szoor et al., 2010) providing an exciting area of metabolic control that could generate wide-ranging changes to the parasites' physiology, adapting them to life in the tsetse midgut.

As these perception and response pathways become characterized and central regulators identified, therapeutic opportunities will certainly emerge. Parasite-specific essential proteins will provide novel drug targets, whereas the density-sensing mechanisms provide opportunities for perturbing signal transduction or manipulating the quorum-sensing signal. This represents an area of considerable interest in microbial therapeutics, since interfering with communication between microbes can generate imaginative therapeutic approaches whereby pathogen signals can be manipulated to block virulence under conditions where resistance is selected against (Brown et al., 2009). Clearly, the developmental events that underpin the pre-adaptation of trypanosomes for life in the tsetse fly provides a rich seam of exciting biology, molecular biology and therapeutic potential that will sustain trypanosome researchers, and excite the wider biological community, for many years to come.

## Author contributions

All authors contributed equally to this manuscript. The relevant sections were authored as follows: Gene expression (Eva Rico), Quorum sensing and signaling (Binny M. Mony), Adaptations at the parasite surface (Federico Rojas), Signaling in the tsetse fly (Balazs Szoor), Drug Screening (Paula MacGregor), Introductory and concluding sections and manuscript assembly (Keith R. Matthews).

### Conflict of interest statement

The authors declare that the research was conducted in the absence of any commercial or financial relationships that could be construed as a potential conflict of interest.
